# Supplementary material for: Does previous circumcision and wealth index influence women’s attitude to discontinue the practice of female genital mutilation and cutting (FGM/C) in Ethiopia?
Source: PLoS One. 2022 Aug 19;17(8):e0272934. doi: 10.1371/journal.pone.0272934 (PMC9390897; doi:10.1371/journal.pone.0272934)
Supplement: Attachment — Submitted filename: Filled Review Form for PONE-D-21-07908 BALDE Mamadou Dioulde 10.02.2022 (2).docx [file pone.0272934.s001.docx]

PLOS ONE

Does previous circumcision and wealth index influencing women’s attitude to

discontinue the practice of female genital mutilation and cutting (FGM/C) in Ethiopia?

--Manuscript Draft--

| Manuscript Number: | PONE-D-21-07908 |
| --- | --- |
| Article Type: | Research Article |
| Full Title: | Does previous circumcision and wealth index influencing women’s attitude to discontinue the practice of female genital mutilation and cutting (FGM/C) in Ethiopia? |
| Short Title: | Does previous circumcision and wealth index influencing discontinuation of FGM in Ethiopia |
| Corresponding Author: | Mohammed Ahmed, MPH  Woldia University  Woldia, ETHIOPIA |
| Keywords: | Female genital mutilation and cutting, previous circumcision, wealth index, attitude, Ethiopia |
| Abstract: | Introduction: The first step toward altering the practice of female genital mutilation is to change attitudes toward it, even though this can be difficult and psychologically painful. Hence, the current study assessed the association between wealth index and women’s attitude to discontinue the practice of FGM/C in Ethiopia using the recent 2016 nationally representative Demographic and Health Survey.  Methods: We extracted the data from the 2016 Ethiopia Demographic and Health Survey data set, which was a cross-sectional study among 6984 weighted samples of women aged 15-49 years. Multivariable logistic regression analysis was performed to verify the hypothesis.  Result: In this study, improved wealth index and women increased level of education results in a higher likelihood of the attitude towards the discontinuation of FGM. Besides, the attitude was lower among circumcised women (AOR: 0.22; 95% CI: 0.150.32), women live in the Afar (AOR: 0.34; 95% CI: 0.22-0.50), Somali (AOR: 0.42; 95% CI: 0.27-0.65), and Dire Dawa region (AOR: 0.51; 95% CI: 0.32-0.83) lowers the attitude towards discontinuation of female genital mutilation.  Conclusion: Empowering women in socioeconomic status and education to have an impact on the attitude to deter female genital mutilation and cutting. Furthermore, designing interventions that address FGM/C practices should focus on circumcised women along with addressing regional variation to have a significant effect on curtailing this harmful traditional practice from Ethiopia. |
| Order of Authors: | Mohammed Ahmed, MPH |
|  | Abdu Seid |
|  | Seada Seid |
| Additional Information: |  |
| Question | Response |
| Financial Disclosure  Enter a financial disclosure statement that describes the sources of funding for the work included in this submission. Review the [submission guidelines](http://journals.plos.org/plosone/s/submission-guidelines#loc-financial-disclosure-statement) for detailed requirements. View published research articles from [PLOS ONE](http://journals.plos.org/plosone/) for specific examples.  This statement is required for submission | The author(s) received no specific funding for this work. |

| and will appear in the published article if the submission is accepted. Please make sure it is accurate. |  |
| --- | --- |
| Unfunded studies Enter: The author(s) received no specific funding for this work. Funded studies Enter a statement with the following details: - Initials of the authors who received each award - Grant numbers awarded to each author - The full name of each funder - URL of each funder website - Did the sponsors or funders play any role in the study design, data collection and analysis, decision to publish, or preparation of the manuscript? - NO - Include this sentence at the end of your statement: The funders had no role in study design, data collection and analysis, decision to publish, or preparation of the manuscript. - YES - Specify the role(s) played. |  |
| * typeset |  |
| Competing Interests  Use the instructions below to enter a competing interest statement for this submission. On behalf of all authors, disclose any [competing interests](http://journals.plos.org/plosone/s/competing-interests) that could be perceived to bias this work—acknowledging all financial support and any other relevant financial or nonfinancial competing interests.  This statement is required for submission and will appear in the published article if the submission is accepted. Please make sure it is accurate and that any funding sources listed in your Funding Information later in the submission form are also declared in your Financial Disclosure statement.  View published research articles from [PLOS ONE](http://journals.plos.org/plosone/) for specific examples. | The authors have declared that no competing interests exist. |

| NO authors have competing interests Enter: The authors have declared that no competing interests exist. Authors with competing interests Enter competing interest details beginning with this statement: I have read the journal's policy and the authors of this manuscript have the following competing interests: [insert competing interests here] |  |
| --- | --- |
| * typeset |  |
| Ethics Statement  Enter an ethics statement for this submission. This statement is required if the study involved:   - Human participants - Human specimens or tissue - Vertebrate animals or cephalopods - Vertebrate embryos or tissues - Field research   Write "N/A" if the submission does not require an ethics statement.  General guidance is provided below. Consult the [submission guidelines](http://journals.plos.org/plosone/s/submission-guidelines#loc-guidelines-for-specific-study-types) for detailed instructions. Make sure that all information entered here is included in the Methods section of the manuscript. | Since it is secondary data analysis from the 2016 EDHS database, ethical clearance for the study is not required. The researchers of this study have maintained the confidentiality of the data after received from the USAID–DHS program. Before the study commencement, consent was obtained from the study participants. |

Format for specific study types

Human Subject Research (involving human

participants and/or tissue)

Give the name of the institutional review

board or ethics committee that approved the

study

•

Include the approval number and/or a

statement indicating approval of this

research

•

Indicate the form of consent obtained

(

written/oral) or the reason that consent was

not obtained (e.g. the data were analyzed

anonymously)

•

Animal Research (involving vertebrate

animals, embryos or tissues)

Provide the name of the Institutional Animal

Care and Use Committee (IACUC) or other

relevant ethics board that reviewed the

study protocol, and indicate whether they

approved this research or granted a formal

waiver of ethical approval

•

Include an approval number if one was

obtained

•

If the study involved

non-human primates

,

add

additional details

about animal welfare

and steps taken to ameliorate suffering

•

If anesthesia, euthanasia, or any kind of

animal sacrifice is part of the study, include

briefly which substances and/or methods

were applied

•

Field Research

Include the following details if this study

involves the collection of plant, animal, or

other materials from a natural setting:

Field permit number

•

Name of the institution or relevant body that

granted permission

•

Data Availability

Authors are required to make all data

underlying the findings described fully

available, without restriction, and from the

time of publication. PLOS allows rare

exceptions to address legal and ethical

concerns. See the

[PLOS Data Polic](http://journals.plos.org/plosone/s/data-availability)

[y](http://journals.plos.org/plosone/s/data-availability)

and

[FA](http://journals.plos.org/plosone/s/data-availability#loc-faqs-for-data-policy)

[Q](http://journals.plos.org/plosone/s/data-availability#loc-faqs-for-data-policy)

for detailed information.

No - some restrictions will apply

| A Data Availability Statement describing where the data can be found is required at submission. Your answers to this question constitute the Data Availability Statement and will be published in the article, if accepted. |  |
| --- | --- |
| Important: Stating ‘data available on request from the author’ is not sufficient. If your data are only available upon request, select ‘No’ for the first question and explain your exceptional situation in the text box. |  |
| Do the authors confirm that all data underlying the findings described in their manuscript are fully available without restriction? |  |
| Describe where the data may be found in full sentences. If you are copying our sample text, replace any instances of XXX with the appropriate details.   - If the data are held or will be held in a public repository, include URLs, accession numbers or DOIs. If this information will only be available after acceptance, indicate this by ticking the box below. For example: All XXX files   are available from the XXX database (accession number(s) XXX, XXX.).   - If the data are all contained within the manuscript and/or Supporting Information files, enter the following: All relevant data are within the manuscript and its Supporting Information files. - If neither of these applies but you are able to provide details of access elsewhere, with or without limitations, please do so. For example:   Data cannot be shared publicly because of [XXX]. Data are available from the XXX Institutional Data Access / Ethics Committee (contact via XXX) for researchers who meet the criteria for access to confidential data.  The data underlying the results presented in the study are available from (include the name of the third party | We used the USAID–DHS program 2016 Ethiopian demographic and health survey data set for this analysis. To request the same or different data for another purpose, a new research project request should be submitted to the DHS program here:  https://dhsprogram.com/data/Access-Instructions.cfm. After receiving permission, the researcher can log in and select the specific data in the format they prefer. |

| and contact information or URL).  • This text is appropriate if the data are owned by a third party and authors do not have permission to share the data.  * typeset |  |
| --- | --- |
| Additional data availability information: |  |

Manuscript	[Click here to access/download;Manuscript;FGM manuscript final.docx](https://www.editorialmanager.com/pone/download.aspx?id=28573704&guid=35599189-fefb-4a73-af13-8fb36544e409&scheme=1)

***Does previous circumcision and wealth index influencing women’s attitude to discontinue the practice of female genital mutilation and cutting (FGM/C) in Ethiopia?***

Mohammed Ahmed^1*^, Abdu Seid^2^, Seada Seid^1^

^1^Department of Public Health, College of Health Science, Woldia University, Woldia, Ethiopia

^2^Department of Midwifery, College of Health Science, Woldia University, Woldia, Ethiopia

**Email address**

^1*^Corresponding author**: abdus3536@gmail.com**

^2^Mohammed Ahmed**: mohaasrar12@gmail.com** ^3^Seada Seid: seadaseid814@gmail.com

# Abstract

**Introduction:** The first step toward altering the practice of female genital mutilation is to change attitudes toward it, even though this can be difficult and psychologically painful. Hence, the current study assessed the association between wealth index and women’s attitude to discontinue the practice of FGM/C in Ethiopia using the recent 2016 nationally representative Demographic and Health Survey.

**Methods:** We extracted the data from the 2016 Ethiopia Demographic and Health Survey data set, which was a cross-sectional study among 6984 weighted samples of women aged 15-49 years**.** Multivariable logistic regression analysis was performed to verify the hypothesis. **Result:** In this study, improved wealth index and women increased level of education results in a higher likelihood of the attitude towards the discontinuation of FGM. Besides, the attitude was lower among circumcised women (AOR: 0.22; 95% CI: 0.15-0.32), women live in the Afar (AOR: 0.34; 95% CI: 0.22-0.50), Somali (AOR: 0.42; 95% CI: 0.27-0.65), and Dire Dawa region (AOR: 0.51; 95% CI: 0.32-0.83) lowers the attitude towards discontinuation of female genital mutilation.

**Conclusion:** Empowering women in socioeconomic status and education to have an impact on the attitude to deter female genital mutilation and cutting. Furthermore, designing interventions that address FGM/C practices should focus on circumcised women along with addressing regional variation to have a significant effect on curtailing this harmful traditional practice from Ethiopia.

**Keywords:** Female genital mutilation and cutting, previous circumcision, wealth index, attitude, Ethiopia

# Background

Female genital mutilation/cutting (FGM/C) is defined as all procedures that involve partial or total removal of the external female genitalia or other injuries to the female genital organs for non-medical reasons(1). The prevalence of FGM/C in Ethiopia has decreased over the past 16 years, dropping from 80% in the 2000 Ethiopia Demographic and Health Survey (EDHS) to 74% in 2005, and 65% in 2016 EDHS(2). However, the prevalence differs across regions; for instance, 99% in Somali, 91% in Afar, 33% in Gambela, and 24% in Tigray(2).

FGM is a hazardous practice that predates all religions and is perpetrated on children for reasons in various cultural and global contexts(3), causing short and long-term adverse health consequences such as hemorrhage, shock, urinary retention, and infertility (4-7). Also, a study done in Ghana showed that FGM was associated with an 8.2% cesarean section rate compared with 6.7% in mothers without FGM(8).

The first step toward shifting the practice of FGM is to change attitudes toward it, even though this can be difficult and psychologically painful(9). In Ethiopia, 21 % percent of women believe that the practice should be continued(2). Studies showed that attitude towards the discontinuation of FGM was attributed to different factors such as male sex (10, 11), urban residence (12), educational attainment (5, 12-14), access to media (12, 13). By contrast, women with positive cultural conceptions of FGM, being Muslim religion follower (12, 13, 15), aged 15–24 years, rural residents, married(13) were less likely to favor its discontinuation

Concerning the influence of circumcision and socioeconomic status on attitudes to FGM, pieces of evidence reported inconsistent results; for instance, a study conducted in Egypt depicted the previous circumcision favors discontinuation (11), but, a study conducted in Ethiopia showed that previous circumcision results in the continuation of FGM(13). Similarly, higher levels of household wealth increased women’s support for discontinuation (15, 16). In some countries, wealth was associated with supporting FGM, and in some the opposite (17).

Previous studies conducted in Ethiopia on determining whether Ethiopian women support the continuation of female genital mutilation(13, 18), was conducted without including indispensable covariates such as wealth index, as the attitude is affected by socioeconomic status.

Therefore, the current study endeavored to fill this gap by investigating the association between wealth index and women’s attitude to discontinue the practice of FGM/C in Ethiopia using the recent 2016 national demographic and health survey data set to eliminate the practice of FGM/C from the nation.

# Methods Data source

The current study utilized the recent 2016 Ethiopia Demographic and Health Surveys (EDHS) data set. A two-stage stratified cluster sampling method was used. A detailed description of the study design and methodology of the survey were founded elsewhere(2). About 15,683 women (15–49 years) were interviewed in the 2016 EDHS, making up response rates of 95%. The analytic sample in this study included 6984 reproductive age women (15- 49 years) after removing cases that had missing values on the variable of interests.

# Study variables

The dependent variable was the attitude towards the discontinuation of female genital mutilation, which was measured by asking the questions “circumcision should continue or be stopped?” The respondents had two options that should continue, and should not continue. The main exposure variables were ever been circumcised(yes or no) and wealth index (a composite index based on the household’s ownership of several consumer items) which had five categories (poorest, poorer, middle richer, and richest) according to the demographic and health survey data set(2).

Variables such as the age of the respondents, religion, marital status, education status, type of residence, access to media, and regions were selected as covariates that affect attitude towards the discontinuation of female genital mutilation.

# Statistical Analysis

SPSS version 21 was used to analyze the data. Frequencies and weighted percentages of study variables were reported. Bivariate analysis using Rao–Scott chi-square test was performed to examine the relationship between attitude towards the discontinuation of female genital mutilation and each of the independent variables and to select potential candidates for the multivariable model. A variable, which had a p-value of < 0.25 in bivariate analysis were entered into a multivariable logistic regression analysis to assess the association between wealth index, previous circumcision and women’s attitude to discontinue the practice of FGM/C by controlling confounders. To declare a statistically significant association, adjusted odds ratios (AOR) with 95% confidence interval (CI) were used.

# Ethics approval and consent to participate

Since it is secondary data analysis from the 2016 EDHS database, ethical clearance for the study is not required. The researchers of this study have maintained the confidentiality of the data after received from the USAID–DHS program. Before the study commencement, consent was obtained from the study participants.

# Results

# Description of the study variables with attitude to discontinuation of FGM

A total of 6984 reproductive-aged women within the EDHS 2016 were included and analyzed. Regarding age, 35.4% of women aged 20-29 years had an attitude toward discontinuation of female genital mutilation. Concerning the circumcision status, 65.8 % of the respondents who supported discontinuation of FGM were ever been circumcised. **(Table 1).**

**Table 1: Univariate and bivariate analysis of the study variables with attitude to female genital mutilation among reproductive-age women in Ethiopia (n=6984).**

| Variables | Category | Overall | Attitude to FGM | | p-value |
| --- | --- | --- | --- | --- | --- |
|  |  |  | Discontinued | Continued |  |
|  |  | n(wt.%) | n(wt.%) | n(wt.%) |  |
| Age | 15-19 | 1523(21.4) | 1225(22.5) | 298(16.4) | 0.004 |
|  | 20-29 | 2547(35.2) | 1992(35.4) | 555(34.4) |  |
|  | 30-39 | 1879(28.3) | 1461(27.3) | 418(32.7) |  |
|  | 40-49 | 1035(15.1) | 789(14.7) | 246(16.6) |  |
| Residence | Urban | 2543(23.4) | 2284(26.5) | 259(9.6) | <0.001 |
|  | Rural | 4441(76.6) | 3183(73.5) | 1258(90.4) |  |
| Religion | Non-Muslim | 5862(76.5) | 4462(75.2) | 1400(82.7) | 0.023 |
|  | Muslim | 1122(23.5) | 1005(24.8) | 117(17.3) |  |
| Marital Status | Never Married | 1942(25.8) | 1674(28.0) | 268(15.4) | <0.001 |
|  | Married | 4333(64.7) | 3209(62.3) | 1124(75.8) |  |
|  | Separated/divorced | 709(9.5) | 584(9.7) | 125(8.8) |  |
| Education | No Education | 3011(45.9) | 1959(41.2) | 1052(67.0) | <0.001 |
|  | Primary | 2325(35.1) | 1953(36.5) | 372(28.9) |  |
|  | Secondary | 1058(12.6) | 985(14.6) | 73(3.7) |  |
|  | Higher | 590(6.4) | 570(7.7) | 20(0.4) |  |
| Wealth index | Poorest | 1605(15.4) | 853(12.2) | 752(29.9) | <0.001 |
|  | Poorer | 862(17.5) | 659(16.5) | 203(22.0) |  |
|  | Middle | 900(19.5) | 730(19.4) | 170(19.8) |  |
|  | Richer | 910(19.9) | 772(20.6) | 138(16.4) |  |
|  | Richest | 2707(27.8) | 2453(31.3) | 254(11.8) |  |
| Access to media | No | 3426(54.1) | 2353(51.1) | 1073(68.0) | <0.001 |
|  | Yes | 3558(45.9) | 3114(48.9) | 444(32.0) |  |
| Ever been  Circumcised | No | 2113(29.4) | 2015(34.2) | 98(7.7) | <0.001 |
|  | Yes | 4871(70.6) | 3452(65.8) | 1419(92.3) |  |
| Region | Tigray | 658(6.4) | 604(7.1) | 54(2.9) | <0.001 |
|  | Afar | 579(0.9) | 226(0.5) | 353(2.9) |  |
|  | Amhara | 739(22.8) | 601(22.9) | 138(22.4) |  |
|  | Oromiya | 876(38.0) | 712(37.1) | 164(41.7) |  |
|  | Somali | 692(3.2) | 312(1.9) | 380(9.4) |  |
|  | Benishangul- Gumuz | 475(1.0) | 431(1.1) | 44(0.5) |  |
|  | SNNPR | 786(20.1) | 670(20.6) | 116(17.6) |  |
|  | Gambela | 322(0.2) | 296(0.2) | 26(0.1) |  |
|  | Harari | 414(0.3) | 350(0.3) | 64(0.2) |  |
|  | Addis Ababa | 894(6.5) | 861(7.7) | 33(1.4) |  |
|  | Dire Dawa | 549(0.6) | 404(0.6) | 145(0.9) |  |

# Association between wealth index and women’s attitude to discontinue the practice of FGM/C in Ethiopia

All the variables were entered into multivariable logistic regression analysis since all the variables had a p-value of <0.25 in bivariate analysis. The odds of attitude towards the discontinuation of female genital mutilation was 1.46 (AOR: 1.46; 95% CI: 1.02-2.01), 1.95 (AOR:1.95; 95% CI: 1.36-2.79), 2.22 (AOR: 2.22; 95% CI: 6.68-31.4), and 2.65 (AOR: 2.65; 95% CI: 1.57-4.46) times higher among women who had a poorer, middle, richer, and richest wealth index quintile, respectively compared to women who were in the poorest quintile. Besides, the odds of attitude towards the discontinuation of female genital mutilation was 1.57 (AOR: 1.57; 95% CI: 1.17-2.12), 3.38 (AOR: 3.38; 95% CI: 1.96-5.83), 14.5(AOR: 14.5; 95% CI: 6.68-31.4) times higher among women attained primary, secondary, and higher education, respectively compared to non educated one.

Also, circumcised women compared to non-circumcised women lower the odds of the attitude towards the discontinuation of female genital mutilation by 78% (AOR: 0.22; 95% CI: 0.150.32). Besides, the odds of the attitude towards the discontinuation of female genital mutilation was lower by 66% (AOR: 0.34; 95% CI: 0.22-0.50) among women living in the Afar region, by 38% (AOR: 0.42; 95% CI: 0.27-0.65) among women living in the Somali region, by 59% (AOR: 0.51; 95% CI: 0.32-0.83) among women live in Dire Dawa compared to Oromiya region. However, the odds of the attitude towards the discontinuation of female genital mutilation was higher among women living in Benishangul- Gumuz by 2.22 times (AOR: 2.22; 95% CI: 1.46-

3.36), and Addis Ababa by 2.13 times (AOR: 2.13; 95% CI: 1.13-4.04) (**Table 2).**

**Table 2: Multivariable analysis using binary logistic regression to identify independent predictors of attitude toward discontinuation of female genital mutilation among reproductive-age women in Ethiopia (n=6984).**

| variables | category | Attitude to discontinuation FGM | |
| --- | --- | --- | --- |
|  |  | COR(95% CI) | AOR(95%CI) |
| Age | 15-19 | 1 | 1 |
|  | 20-29 | 0.75(0.57-0.97) | 1.08(0.77-1.52) |
|  | 30-39 | 0.61(0.45-0.81) | 1.25(0.82-1.94) |
|  | 40-49 | 0.65(0.48-0.86) | 1.32(0.89-1.94) |
| Residence | Urban | 3.39(2.49-4.60) | 1.01(0.62-1.63) |
|  | Rural | 1 | 1 |
| Religion | Non-Muslim | 1 | 1 |
|  | Muslim | 1.58(1.06-2.36) | 1.45(0.98-2.13) |
| Marital Status | Never Married | 1 | 1 |
|  | Married | 0.45(0.35-0.58) | 0.96(0.67-1.36) |
|  | Separated/Divorced | 0.61(0.42-0.87) | 1.08(0.69-1.69) |
| Education | No education | 1 | 1 |
|  | Primary | 2.05(1.63-2.58) | 1.57(1.17-2.12)* |
|  | Secondary | 6.42(4.03-10.2) | 3.38(1.96-5.83)* |
|  | Higher | 30.5(16.3-57.5) | 14.5(6.68-31.4)* |
| Wealth Index | Poorest | 1 | 1 |
|  | Poorer | 1.83(1.33-2.53) | 1.46(1.02-2.01)* |
|  | Middle | 2.40(1.75-3.28) | 1.95(1.36-2.79)* |
|  | Richer | 3.08(2.27-4.17) | 2.22(1.55-3.19)* |
|  | Richest | 6.48(4.65-9.03) | 2.65(1.57-4.46)* |
| Access to media | No | 1 | 1 |
|  | Yes | 2.04(1.64-2.53) | 0.85(0.65-1.13) |
| Ever been Circumcised | No | 1 | 1 |
|  | Yes | 0.16(0.11-0.23) | 0.22(0.15-0.32)* |
| Region | Tigray | 2.75(1.65-4.55) | 1.47(0.84-2.58) |
|  | Afar | 0.19(0.14-0.28) | 0.34(0.22-0.50)* |
|  | Amhara | 1.14(0.78-1.66) | 0.96(0.68-1.37) |
|  | Oromiya | 1 | 1 |
|  | Somali | 0.22(0.15-0.33) | 0.42(0.27-0.65)* |
|  | Benishangul- Gumuz | 2.27(1.43-3.61) | 2.22(1.46-3.36)* |
|  | SNNPR | 1.31(0.83-2.06) | 0.95(0.63-1.44) |
|  | Gambela | 2.91(1.82-4.64) | 1.22(0.72-2.07) |
|  | Harari | 1.26(0.80-1.99) | 1.02(0.65-1.57) |
|  | Addis Ababa | 6.30(3.93-10.1) | 2.13(1.13-4.04)* |
|  | Dire Dawa | 0.74(0.49-1.10) | 0.51(0.32-0.83)* |

*-showed significant association with AOR with a 95% confidence interval at a p-value of < 0.05

**Discussion**

The current study investigated that women who get a better wealth index augment the likelihood of the attitude to discontinuation of FGM/C in Ethiopia. This finding is supported by studies conducted in Guinea(16) and other countries(15). This is due to empowering women economically leads to resisting the practice through fostering decision-making power (19).

This study also adds to the literature that educated women, previous circumcision, and regional variation were significant predictors of women’s attitude to discontinue the practice of FGM/C in Ethiopia. Educational attainment of the women results in a higher likelihood of the attitude to discontinuation of female genital cutting. This finding is consistent with studies conducted in different places (5, 12-14). This may be explained as educated women may be equipped with the knowledge to evaluate their beliefs about traditional practices, and it provides women with financial independence and empowerment to liberate themselves from harmful practices. Furthermore, the opinions of educated women are less likely to be influenced and shaped by traditions.

This study showed that circumcised women were less likely to favor the discontinuation of FGM compared with uncircumcised women. This finding is in line with a previous study conducted in Ethiopia(13), This can be explained as growing up with traditional values can instill a deep meaning and purpose into the practice of FGM. The women who are circumcised may have experienced the benefits of the practice such as recognition and respect. But, the current finding is contrary to a study conducted in Egypt (11), which showed previous circumcision results in discontinuation of FGM/C. This variation from the present study is due to differences in the study participants in which the study conducted in Egypt was among medical students whereas the current study is among reproductive-age women reside in the community. Since they were medical students they easily anticipate the health consequences of FGM thereby increased their positive attitude towards the discontinuation of the FGM/C.

Besides, the odds of the attitude towards the discontinuation of female genital mutilation were lower among women who live in Afar, Somali, and Dire Dawa. However, the attitude towards the discontinuation of female genital mutilation was higher among women living in Benishangul- Gumuz, and Addis Ababa. This finding is consistent with previous studies conducted in Ethiopia (20, 21). This could be due to significant Spatio-temporal variation of FGM practice across the country (20).

# Strength and limitation of the study

Although findings in this study are useful for policy, there are some noteworthy limitations. For example, the data were collected from secondary data and our study may not be devoid of the shortcomings associated with this approach. Notwithstanding these limitations, our study is one of the few that have contributed in the context of the association between wealth index and women’s attitude to discontinue the practice of FGM/C in Ethiopia, as no other studies were done in the country using the recent 2016 national representative data set up to date.

# Conclusions

The present study revealed that women’s wealth index, educated women, previous circumcision, and regional variation significantly predict women’s attitude to discontinue the practice of FGM/C in Ethiopia. Therefore, efforts need to be done in promoting women’s socioeconomic status concurrently empowering women in education. Furthermore, designing interventions that address FGM/C practices should focus on circumcised women along with addressing regional variation to have a significant effect on curtailing this harmful traditional practice from Ethiopia.

# Acknowledgments

We would like to express gratitude to the USAID–DHS program for offering the 2016 Ethiopia Demographic and Health Survey data set.

# Author Contributions

All authors made a significant contribution to the work reported in all areas; took part in drafting, revising, or extremely reviewing the article; gave a final endorsement of the version to be published; have agreed on the journal to which the article has been submitted; and agree to be responsible for all aspects of the work.

**Competing interests:** We confirm that this research is our original paper and that there is no conflict of interest in this work.

# Data availability statement

We used the USAID–DHS program 2016 Ethiopian demographic and health survey data set for this analysis. To request the same or different data for another purpose, a new research project request should be submitted to the DHS program here: [**https://dhsprogram.com/data/AccessInstructions.cfm**.](https://dhsprogram.com/data/Access-Instructions.cfm) After receiving permission, the researcher can log in and select the specific data in the format they prefer.

**Funding**

There is no funding to report

# Reference

1. WHO. Eliminating female genital mutilation: an interagency statement-OHCHR, UNAIDS,

UNDP, UNECA, UNESCO, UNFPA, UNHCR, UNICEF, UNIFEM, WHO: World Health Organization; 2008.

2. ICF CSACEa. Ethiopia Demographic and Health Survey. Addis Ababa, Ethiopia, and Rockville, Maryland, USA: CSA and ICF.2016.
3. Ndasi DM, Adusei-Asante K. Female Genital Mutilation/Cutting: A Conceptual Discussion. Advances in Social Sciences Research Journal. 2019;6(11):232-46.
4. Almroth L, Elmusharaf S, El Hadi N, Obeid A, El Sheikh MA, Elfadil SM, et al. Primary infertility after genital mutilation in girlhood in Sudan: a case-control study. The Lancet. 2005;366(9483):385-91.
5. Johnson OE, Okon RD. Perception and practice of female genital cutting in a rural community in southern Nigeria. African journal of reproductive health. 2012;16(4):132-9.
6. Aziz F. Gynecologic and obstetric complications of female circumcision. International Journal of Gynecology & Obstetrics. 1980;17(6):560-3.
7. Makhlouf Obermeyer C. The consequences of female circumcision for health and sexuality: an update on the evidence. Culture, health & sexuality. 2005;7(5):443-61.
8. Oduro A, Ansah P, Hodgson A, Afful T, Baiden F, Adongo P, et al. Trends in the prevalence of female genital muti-lation and its effect on delivery outcomes in the kassena-nankana district of northern Ghana. Ghana medical journal. 2006;40(3).
9. Lien I-L, Schultz J-H. Internalizing knowledge and changing attitudes to female genital cutting/mutilation. Obstetrics and gynecology international. 2013;2013.
10. Abathun AD, Sundby J, Gele AA. Attitude toward female genital mutilation among Somali and Harari people, Eastern Ethiopia. International journal of women's health. 2016;8:557-69.
11. Abolfotouh SM, Ebrahim AZ, Abolfotouh MA. Awareness and predictors of female genital mutilation/cutting among young health advocates. International journal of women's health. 2015;7:259-69.
12. Dalal K, Lawoko S, Jansson B. Women's attitudes towards discontinuation of female genital mutilation in Egypt. Journal of injury & violence research. 2010;2(1):41-5.
13. Saba W. Masho LM. Factors determining whether Ethiopian women support continuation of female genital mutilation. International Journal of Gynecology and Obstetrics. 2009(107):232–5.
14. Ashimi AO, Amole TG. Perception and attitude of pregnant women in a rural community northwest Nigeria to female genital mutilation. Archives of gynecology and obstetrics. 2015;291(3):695-700.
15. Dalal K, Kalmatayeva Z, Mandal S, Ussatayeva G, Lee MS, Biswas A. Adolescent girls' attitudes toward female genital mutilation: a study in seven African countries. F1000Research. 2018;7:343.
16. A.J. Gage R. Van Rossem ~~RVR~~. Attitudes toward the discontinuation of female genital cutting among men and women in Guinea. International Journal of Gynecology and Obstetrics. 2006(92):92 — 6.
17. Sipsma HL, Chen PG, Ofori-Atta A, Ilozumba UO, Karfo K, Bradley EH. Female genital cutting: current practices and beliefs in western Africa. Bulletin of the World Health Organization. 2012;90(2):120-7f.
18. Fikrie Z. Factors Associated with Perceived Continuation of Females' Genital Mutilation among Women in Ethiopia. Ethiopian journal of health sciences. 2010;20(1):49-53.
19. Toubia NF, Sharief EH. Female genital mutilation: have we made progress? International journal of gynaecology and obstetrics: the official organ of the International Federation of Gynaecology and Obstetrics. 2003;82(3):251-61.
20. Tesema GA, Agegnehu CD, Teshale AB, Alem AZ, Liyew AM, Yeshaw Y, et al. Trends and Spatio-temporal variation of female genital mutilation among reproductive-age women in Ethiopia: a Spatio-temporal and multivariate decomposition analysis of Ethiopian demographic and health surveys. BMC public health. 2020;20(1):719.
21. Setegn T, Lakew Y, Deribe K. Geographic Variation and Factors Associated with Female Genital Mutilation among Reproductive Age Women in Ethiopia: A National Population Based Survey. PloS one. 2016;11(1):e0145329.
