# Supplementary material for: Does previous circumcision and wealth index influence women’s attitude to discontinue the practice of female genital mutilation and cutting (FGM/C) in Ethiopia?
Source: PLoS One. 2022 Aug 19;17(8):e0272934. doi: 10.1371/journal.pone.0272934 (PMC9390897; doi:10.1371/journal.pone.0272934)
Supplement: Attachment — Submitted filename: Response to reviewers.docx [file pone.0272934.s002.docx]

Thank you very much for PLOS one editorial office, academic editors, as well as reviewers of this manuscript entitled ***Does previous circumcision and wealth index influence women’s attitude to discontinue the practice of female genital mutilation in Ethiopia?*** For their astonished effort.

The written documents below explained point by point response for respective editor and reviewer’s comment.

**Editor comments and author response**

During your revisions, please note that a simple title correction is required: The grammar choice of 'influencing' in the title is not correct, one suggested edit to the title could be - 'Does previous circumcision and wealth index influence women’s attitude to discontinue the practice of female genital mutilation and cutting (FGM/C) in Ethiopia?'.

**Author response: Thank you so much. The authors amended the manuscript based on the reviewer comments and included in the revised manuscript.**

**Reviewer 1 comments and authors response:**

Reviewer #1: 

General comment on the manuscript.
FGM/C and FGM acronyms and circumcision were inconsistently used in the manuscript. Please make up your mind on the one you want to use and be consistent with its use throughout the manuscript. You also need to clarify that female genital mutilation/cutting is also known as female circumcision so that this becomes clearer for lay global audience. The manuscript will benefit from editing from primary speakers of English language.

**Author response: Thank you so much. The authors amended the manuscript based on the reviewer comments and included in the revised manuscript.**
Title: This looks clumsy and unclear. I suggest that you write it out in a clear statement. If you insist on presenting it as a question, kindly revise to ensure clarity.

**Author response: The authors amended the manuscript based on the reviewer comments and included in the revised manuscript.**
Abstract
Conclusion: The 1st sentence is not complete. Kindly revise

**Author response: The authors amended the manuscript based on the reviewer comments and included in the revised manuscript.**
Background
As previously indicated, you need clearly clarify female genital mutilation/cutting is also known as female circumcision so that this becomes clearer for lay global audience. For example, in paragraph 4, 1st sentence and the entire paragraph you used circumcision for the first time despite previous use of female genital mutilation/cutting. Be consistent throughout the manuscript.

**Author response: The authors amended the manuscript based on the reviewer comments and included in the revised manuscript.**
Methodology
Data source: 3rd sentence “A detailed description… were founded elsewhere (2)” Do you mean were reported somewhere? How did you arrive at your sample size? Any statistical power analysis?
Study variables: You stated that the wealth index were five, however, you mentioned 4- poorest, poorer, middle, richer, and richest. Please check and amend.

**Author response: The authors amended the manuscript based on the reviewer comments and included in the revised manuscript.** In the 2016 EDHS, 15,683 women (15–49 years) were interviewed, For studying FGM, after excluding cases with missing values on the variables of interests, the analytic sample in this study consisted of 6984 reproductive age women (15- 49 years).

Ethics Analysis
How did you contact and obtain consent from 6984 women of reproductive age whose data you used? This needs to be clearly presented.
**Author response: The authors amended the manuscript based on the reviewer comments and included in the revised manuscript.**
Results
Well presented, thank you.

Discussion
You stated in your findings that “Also, circumcised women compared to non-circumcised women lower the odds of the attitude towards the discontinuation of female genital mutilation by 78%” suggesting that women with FGM/C are more likely to want to continue the practice. However, in the 2nd paragraph of your discussion, you included previous FGM/C as a predictor for discontinuing the practice. Please check and amend.

**Author response: The authors amended the manuscript based on the reviewer comments and included in the revised manuscript.**

Your conflicting discussion of your finding is further depicted in the 3rd paragraph where the content here is different to that of paragraph 2 with reference previous FGM/C and attitude to continue or discontinue FGM/C. Please check and amend.

In general, thank you for this manuscript. Best wishes.

**Author response: The authors amended the manuscript based on the reviewer comments and included in the revised manuscript.**

**Reviewer 2 comments and author response**

Reviewer #2: Corrections about References (See revised Manuscript)

**Author response: The authors amended the manuscript based on the reviewer comments and included in the revised manuscript.**

Reviewer #3: The topic of what explains the persistence of FGC, and attitudes in support of FGC, is an important one. The authors use one survey in one country to examine the correlation between FGC attitudes and wealth and education. Unfortunately I do not see how this piece adds to our knowledge of the perpetuation of FGC above and beyond works that have already been published. The authors note that there are some mixed results regarding socioeconomic status and the practice of FGC. However this study does not help us resolve or make sense of these conflicting results but rather adds another case, which has also been included in past studies. It does not attempt to help us theoretically understand the reason for these conflicting results or present results that help in this regard either. As such, I cannot recommend the article for publication as I do not see it contributing new knowledge to the study of FGC, however I commend the authors for their effort. Perhaps including more countries and over a longer period of time, both of which are possible with the DHS data, could help. Note however that a number of scholars have also already done this

**Author response: There was no study conducted so far in relation to wealth index and attitude to discontinue FGM in Ethiopia. That is why we eager to undertake this study.**

**Thank you**
