# Supplementary material for: Does previous circumcision and wealth index influence women’s attitude to discontinue the practice of female genital mutilation and cutting (FGM/C) in Ethiopia?
Source: PLoS One. 2022 Aug 19;17(8):e0272934. doi: 10.1371/journal.pone.0272934 (PMC9390897; doi:10.1371/journal.pone.0272934)
Supplement: Attachment — Submitted filename: Response to reviewers.docx [file pone.0272934.s003.docx]

Thank you very much for PLOS one editorial office, academic editors, as well as reviewers of this manuscript entitled **Does previous circumcision and wealth index influence women’s attitude to discontinue the practice of female genital mutilation and cutting (FGM/C) in Ethiopia?.**

The written documents below explained point by point response for respective reviewer and editor comments.

**Reviewer 1 comments and authors response:**

Comment 1: the reviewer raised a concern that the manuscript still requires extensive copyediting. We suggest you thoroughly copyedit your manuscript for language usage, spelling, and grammar.

**Author response**: the authors addressed all of the issue raised by the reviewer in the revised manuscript

**Editor comments and author response**

Please review your reference list to ensure that it is complete and correct. If you have cited papers that have been retracted, please include the rationale for doing so in the manuscript text, or remove these references and replace them with relevant current references. Any changes to the reference list should be mentioned in the rebuttal letter that accompanies your revised manuscript. If you need to cite a retracted article, indicate the article’s retracted status in the References list and also include a citation and full reference for the retraction notice.

**Author response**: I have reviewed the references in the manuscript and I did not found retracted articles.

**Thank you for reviewers and editors (staff as well as academic editors) for their extensive and constructive comments throughout the process**
