# Supplementary material for: Does previous circumcision and wealth index influence women’s attitude to discontinue the practice of female genital mutilation and cutting (FGM/C) in Ethiopia?
Source: PLoS One. 2022 Aug 19;17(8):e0272934. doi: 10.1371/journal.pone.0272934 (PMC9390897; doi:10.1371/journal.pone.0272934)
Supplement: Attachment — Submitted filename: Abstract - ONE Edit.docx [file pone.0272934.s004.docx]

**Introduction:** Understanding women’s attitudes towards female genital mutilation is an important step towards eliminating this practice. We used the 2016 Ethiopia Demographic and Health Survey (EDHS) data set to examine the relationship between wealth index and previous history of circumcision on women's opinions whether female genital mutilation (FGM) should be continued or stopped in Ethiopia.

**Methods:** Data from 6984 women aged 15-49 years were extracted from the 2016 EDHS data set. Multivariable logistic regression analysis was performed to analyse the data.

**Result:** In this study, women with a higher level of education and wealth index were more likely to support the cessation of FGM. However, circumcised women (AOR: 0.22; 95% CI: 0.15-0.32), women from the Afar region (AOR: 0.34; 95% CI: 0.22-0.50), Somali region (AOR: 0.42; 95% CI: 0.27-0.65), and Dire Dawa region (AOR: 0.51; 95% CI: 0.32-0.83) were less likely to support discontinuation of FGM.

**Conclusion:** The present study revealed that wealth index, education level, history of circumcision, and regional variation are associated with women’s attitude towards discontinuation of the practice of FGM in Ethiopia. Empowering women in terms of socioeconomic status and education can change attitudes and might help prevent female genital mutilation in the future. Furthermore, interventions targeting FGM practices should focus on regional variance in order to have a meaningful impact on reducing this harmful cultural practice in Ethiopia.
